# The Insight into Obstetric Care near the Front Line in Kharkiv

**DOI:** 10.15388/Amed.2022.29.2.10

**Published:** 2022-06-29

**Authors:** Igor Lakhno

**Affiliations:** Kharkiv Medical Academy of Postgraduate Education, Kharkiv, Ukraine

**Keywords:** pregnancy, obstetric care, military medicine

## Abstract

**Objectives:**

The invasion of Russian troops into independent Ukraine has changed the humanitarian situation in the Kharkiv region. The paper is focused on the peculiarities and issues of the management of labor and delivery near the front line.

**Materials and methods:**

Totally 2030 patients were enrolled in the study. 1410 women who delivered in the second half of 2021 were enrolled in Group I. 620 women who delivered in the first half of 2022 were observed in Group II. Some patients from Group II were used for the investigation of the comparative situation during first months of 2022. 85 women who delivered in January were included in Subgroup IIA. 94 women who have completed their pregnancies and delivered in February were observed in Subgroup IIB. 67 case histories of deliveries which occurred during March were united in Subgroup IIC.

**Results:**

The level of anemia, uterine contractile activity in labor abnormalities, and episiotomies were surprisingly lower during wartime in March. The obtained results showed an almost similar level of obstetric complications, maternal comorbidities, and interventions during labor and delivery in all groups. The stressed environment was a possible reason for the elevation of cardiovascular disease and endocrine disorders.

**Conclusion:**

The study did not reveal any significant changes in the structure of maternal pathologies and obstetric complications during the first months of wartime near the front line. But later the levels of cardiovascular disease and endocrine disorders were found to be elevated.

## Santrauka.

**Tikslai:**

Rusijos kariuomenės įsiveržimas į nepriklausomą Ukrainą pakeitė humanitarinę situaciją Charkivo srityje. Šiame straipsnyje nagrinėjama gimdymo ypatybės pafrontės teritorijose ir kylančios problemos.

**Medžiaga ir metodai:**

Tyrime dalyvavo 2 030 pacienčių. 1 410 moterų, gimdžiusių antrojoje 2021 metų pusėje, sudarė pirmą tyrimo grupę (G I), 320 moterų, kurios gimdė pirmojoje 2022 metų pusėje, priskirtos antrai grupei (G II). Kai kurios pacientės iš antros grupės taip pat buvo įtrauktos į lyginamąjį situacijos per pirmuosius 2022 metų mėnesius tyrimą. 2022 metų sausį gimdžiusios 85 moterys buvo įtrauktos į pogrupį IIA. 94 moterys, kurių nėštumo laikotarpis baigėsi ir kurios gimdė 2022 metų vasarį, pateko į pogrupį IIB. 67 gimdymo atvejai 2022 metų kovą priskirti pogrupiui IIC.

**Rezultatai:**

Stebėtina, tačiau ryški anemija, gimdos susitraukimo veikla sudėtingais gimdymo atvejais ir epiziotomijos atvejai karo laikotarpiu kovo mėnesį buvo retesni. Gauti rezultatai atskleidžia labai panašų visų tiriamųjų grupių akušerinių komplikacijų, gimdyvėms pasireiškiančių sunkių gretutinių ligų ir chirurginių gimdymo intervencijų kiekį. Tikėtina, kad didesnį širdies ir kraujagyslių ligų bei endokrininių sutrikimų kiekį lėmė stresinė aplinka.

**Išvados:**

Tyrimas neatskleidė jokių esminių motinystės patologijų ar akušerinių komplikacijų struktūros pokyčių per pirmuosius mėnesius nuo karo pradžios netoli fronto linijos esančiose teritorijose. Tačiau vėliau buvo nustatytas padidėjęs širdies ir kraujagyslių ligų bei endokrininių sutrikimų lygis.

## Introduction

The invasion of Russian troops into independent Ukraine has changed the humanitarian situation in the Kharkiv region. Thus, all branches of the health care system have been modified in order to provide the population near the front line a qualified and highly-specialized medicine. The female reproductive system has been created by nature itself for a safe birth. The spontaneous onset of labor is a culmination of healthy pregnancy [1]. The mental stress and a continual threat of physical damage have a negative effect on maternal health [2-4].

Two options are known for the population of the regions located close to the area of the armed conflict. Some people ought to leave their homes becoming refugees. The problem of obstetric care in refugees is well known [5]. Several studies have shown an increased level of maternal and perinatal complications in such people [6]. But some pregnant women persist to stay in their native cities. They want to have a safe birth in their local maternal clinics under bombardment despite the continual sound of explosions [7, 8]. Kharkiv municipal perinatal center is located in Saltivka near the border of the city. The staff of the maternal clinic had made significant efforts to follow all current findings and clinical protocols for obstetric care during the war [9-11]. The paper is focused on the peculiarities and issues of the management of labor and delivery near the front line.

## Materials and Methods

A retrospective observational study was performed. The report of delivery unit activity and case histories of deliveries for the six months of the year 2022 and the previous six months of the year 2021 were analyzed. Totally 2030 patients were enrolled in the study. The inclusion criteria were pregnant women who delivered during this period. The exclusion criteria were all case of spontaneous or artificial pregnancy termination before 22 week of gestation. All patients who met the inclusion criteria gave informed consent to participate in the investigation. All ladies were divided into two groups. 1410 women who delivered in the second half of 2021 were enrolled in Group I. 620 women who delivered in the first half of 2022 were observed in Group II. Some patients from Group II were used for the investigation of the comparative situation during first months of 2022. 85 women who delivered in January were included in Subgroup IIA. 94 women who have completed their pregnancies and delivered in February were observed in Subgroup IIB. 67 case histories of deliveries which occurred during March were united in Subgroup IIC.

The study was done by examining the patient records of pregnant women in a hospital computer automation system. All available data including anamnesis, biometry, the variables of births, the level of maternal medical complications and obstetric interventions in labor and delivery were analyzed. We have followed the recommendations for the care of pregnant women in disaster setting [12]. The ACOG criteria for pre-eclampsia, anemia, gestational diabetes mellitus, and poor progress of labor were used [13-18].

Statistical analysis was performed with the Statistical Package for the Social Sciences (SPSS for Windows, Version 25.0, Chicago, IL, USA) program. The Chi-square test or Fischer’s exact test (for 2x2 tables only) was used as a test of significance for qualitative data. A p-value of less than 0.05 was accepted as statistically significant.

## Results

The variables of age were not significantly different in Group I and Group II: 25.3±4.1 years and 25.0±4.6 years (p>0.05). The body mass index was 25.9±4.4 in Group I and 26.1±5.0 in Group II (p>0.05). The average age of the study population was not different in all subgroups: 24.6±4.1 years, 25.1±4.8 years, and 24.9±3.9 years, respectively, in Subgroup IIA, Subgroup IIB, and Subgroup IIC (p>0.05). The parity indices were in these subgroups, respectively: 1.5±0.4 and 1.6±0.5 (p>0.05). The variables of the body mass index were almost similar in Subgroup IIA, Subgroup IIB, and Subgroup IIC: 26.2±4.3, 25.9±3.2, and 26.4±5.6 (p>0.05). The indices of parity in the same subgroups were, respectively: 1.3±0.2, 1.4±0.3, and 1.3±0.3 (p>0.05). Thus, the homogeneity of the study population accordingly to their ages, anthropometric variables, and parity were detected.

The total quantity of deliveries was found to be decreased from the beginning of the war (Table 1). The minimal number of births was registered in March. There were several multiple pregnancies over three months’ period. The level of normal deliveries was not different in all study groups. The level of presence of partners during labor was almost stable with an insignificant reduction in March. The only stillbirth occurred in February. The rate of Cesarean sections was similar in all groups. The number of breech births was not different amongst all study groups. Interestingly, 2 cases of breech delivery in February and 2 cases in March were found to be appropriate for vaginal birth.

**Table 1.** The variables of births in the study population

Variables	Subgroup IIA (January)	Subgroup IIB (February)	p_1_	Subgroup IIC (March)	p_1_; p_2_	Totally for 3 months
Number of births	85	94		67		246
Preterm birth (%)	10 (11.8 %)	6 (6.3 %)	p_1_=0.3018	2 (2.9 %)	p_1_= 0.0749; p_2_= 0.4742	18 (7.3 %)
Multiple birth (%)	1 (1.2 %)	-		1 (1.5 %)		2 (0.8 %)
Normal deliveries (%)	47 (55.3 %)	61 (64.9 %)	p_1_= 0.5425	38 (56.7 %)	p_1_=1.0000; p_2_= 0.6963	146 (59.3 %)
Partnership in labor (%)	50 (58.8 %)	63 (67.0 %)	p_1_= 0.6306	27 (40.3 %)	p_1_= 0.2035; p_2_=0.0777	140 (56.9 %)
Livebirths	86	93		68		247
Stillbirths (%)	-	1 (1.1 %)		-		1 (0.4 %)
Cesarean sections (%)	32 (37.6 %)	33 (35.1 %)	p_1_=0.8850	29 (43.2 %)	p_1_= 0.6513; p_2_=0.5467	94 (38.2 %)
Breech births (%)	3 (3.5 %)	6 (6.4 %)	p_1_=0.5054	2 (3.0 %)	p_1_=1.000; p_2_= 0.4742	11 (4,5 %)
Breech births via Cesarean sections (%)	3 (100 %)	4 (66.7 %)	p_1_=1.000	-		7 (63.6 %)

p_1_ – the difference compared to Subgroup IIA according to χ^2^;

p_2_ – the difference compared to Subgroup IIB according to χ^2^.

The incidence of preterm placental abruption was not significant (Table 2). Such cases were not registered in March. The maximal level of pre-eclampsia was found in February. The insignificant reduction of pre-eclampsia was registered in March. The manifestation of urinary tract infection was not different during peace and wartime. The findings of the insignificantly decreased incidence of anemia in Group II and Group III were not logical. The most disseminated medical problems during pregnancy (cardiovascular disease and endocrine disorders) were found to have similar levels of manifestation in all study groups. The highest statistically significant rate of abnormal uterine contractile activity in labor was detected in February. A slight insignificant reduction of poor progress of labor was found in Group III. The only case of obstetrical hemorrhage occurred in January. The hysterectomy procedure was performed due to placental invasion in the area of the uterine scar. The statistically insignificant tendency to the reduction of episiotomies rate during wartime was detected. Two cases of the manual uterine revision due to retained placental tissue were registered in Group II and Group III. The soft vacuum for fetal extraction was used once in each study group.

The variable of birth did not change even during six months (Table 3). The only significantly different index was partnership in labor. The number of birth was decreased. But the level of livebirth and Cesarean delivery was similar.

**Table 2.** The level of maternal medical complications and obstetric interventions in labor and delivery

Variables	Subgroup IIA (January) N=85	Subgroup IIB (February) N=94	p_1_	Subgroup IIC (March) N=67	p_1_; p_2_	Totally for 3 months N=246
Preterm placental abruption (%)	3 (3.5 %)	2 (2.1 %)	p_1_= 0.6713	-		5 (2.0 %)
Pre-eclampsia (%)	2 (2.4 %)	8 (8.5 %)	p_1_= 0.1112	1 (1.5 %)	p_1_= 1.0000; p_2_= 0.0873	11 (4.5 %)
Gestational pyelonephritis (%)	2 (2.4 %)	6 (6.4 %)	p_1_=0.28811	1 (1.5 %)	p_1_=1.0000; p_2_= 0.2432	9 (3.7 %)
Anemia (%)	10 (11.8 %)	6 (6.4 %)	p_1_=0.3018	3 (4.5 %)	p_1_= 0.2413; p_2_= 0.7380	19 (7.7 %)
Cardiovascular disease (%)	2 (2.4 %)	6 (6.4 %)	p_1_= 0.2881	-		8 (3.3 %)
Endocrine disorders (%)	8 (9.4 %)	5 (5.3 %)	p_1_= 0.3958	5 (7.5 %)	p_1_= 0.2413; p_2_= 0.7772	18 (7.3 %)
Poor progress of labor (%)	1 (1.2 %)	10 (10.6 %)	p_1_= 0.0132	5 (7.5 %)	p_1_= 0.0932; p_2_= 0.5944	16 (6.5 %)
Hemorhage (%)	1 (1.2 %)	-		-		1 (0.4 %)
Hysterectomy (%)	1 (1.2 %)	-		-		1 (0.4 %)
Episiotomy (%)	11 (13.0 %)	10 (10.6 %)	p_1_= 0.8181	2 (3.0 %)	p_1_= 0.0753; p_2_= 0.0736	23 (9.3 %)
Manual uterine revision (%)	-	1 (1.1 %)		1 (1.5 %)		2 (0.8 %)
Soft vacuum for fetal exctraction (%)	1 (1.2 %)	1 (1,1 %)		1 (1.5 %)		3 (1.2 %)

p_1_ – the difference compared to Subgroup IIA according to χ^2^;

p_2_ – the difference compared to Subgroup IIB according to χ^2^.

**Table 3.** The variables of births in the study population during 6 months of 2021 and 2022

Variables	Group I (6 months of 2021)	Group II (6 months of 2022)	p
Number of births	1410	620	
Preterm birth (%)	113 (8.0 %)	43 (6.9 %)	p=0.4704
Multiple birth (%)	34 (2.4 %)	14 (2.3 %)	p=1.0000
Normal deliveries (%)	773 (54.8 %)	356 (57.4 %)	p=0.5739
Partnership in labor (%)	931 (66.0 %)	311 (50.2 %)	p=0.0007*
Livebirths	1434	631
Stillbirths (%)	11 (0.8 %)	3 (0.5 %)	p=0.5710
Cesarean sections (%)	513 (36.4 %)	233 (37.6 %)	p=0.7454
Breech births (%)	63 (4.5 %)	30 (4.8 %)	p=0.7307
Breech births via Cesarean sections (%)	49 (77.8 %)	22 (73.3 %)	p=1.0000

p – the difference compared to Group I according to χ^2^;

* – the difference is statistically significant (p<0.05).

**Table 4.** The level of maternal medical complications and obstetric interventions in labor and delivery during 6 months of 2021 and 2022

Variables	Group I (6 months of 2021) N=1410	Group II (6 months of 2022) N=620	p
Preterm placental abruption (%)	37 (2.6 %)	11 (1.8 %)	p= 0.3402
Pre-eclampsia (%)	82 (5.8 %)	24 ( 3.9 %)	p= 0.1028
Gestational pyelonephritis (%)	41 (2.9 %)	16 (2.6 %)	p= 0.7715
Anemia (%)	100 (7.1 %)	42 (6.8 %)	p= 0.8508
Cardiovascular disease (%)	15 (1.1 %)	15 (2.4 %)	p= 0.0279*
Endocrine disorders (%)	69 (4.9 %)	49 (7.9 %)	p= 0.0141*
Poor progress of labor (%)	95 (6.7 %)	43 (6.9 %)	p= 0.9240
Hemorhage (%)	21 (1.5 %)	8 (1.3 %)	p= 0.8407
Hysterectomy (%)	3 (0.2 %)	1 (0.2 %)	p= 1.0000
Episiotomy (%)	163 (11.6 %)	62 (10.0 %)	p= 0.4001
Manual uterine revision (%)	17 (1.2 %)	9 (1.5 %)	p= 0.6707
Soft vacuum for fetal exctraction (%)	10 (0.7 %)	4 (0.6 %)	p= 1.0000

p – the difference compared to Group I according to χ^2^;

* – the difference is statistically significant (p<0.05).

The variables of cardiovascular disease and endocrine disorders were higher in Group II (Table 4). All other indices of gestational pathologies, complications in labor or postpartum, and obstetric interventions were not different. Therefore, the obstetric care in Kharkiv was stable.

The obtained results showed an almost similar level of obstetric complications, maternal comorbidities, and interventions during labor and delivery in all groups. Therefore, the obvious border between peace and wartime in their projection on maternal health and obstetric complication was not detected either within the first months or even later.

## Discussion

**Figure 1.**
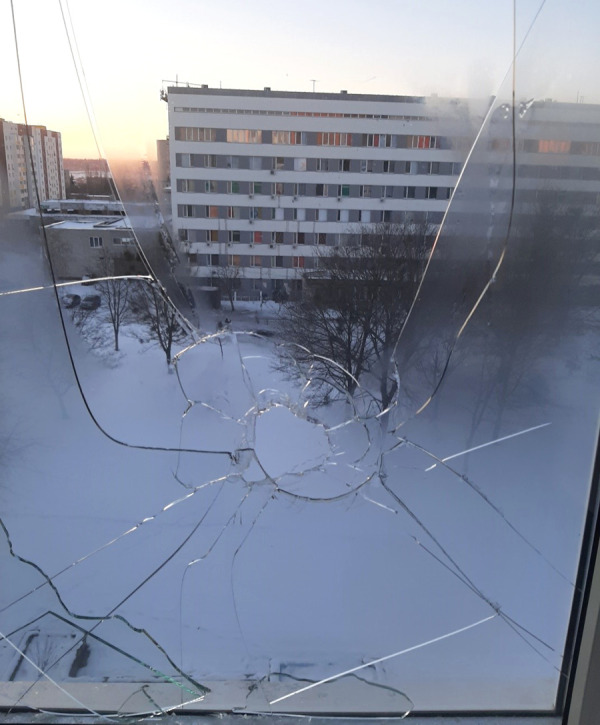
A view through the damaged window from the operative theatre

The main issue in the conflict area is the feeling of being in an unsafe environment and unprotected [19]. Our pregnant women demonstrated courage and a brave spirit. They stayed home and delivered babies in their local perinatal center. They were aware of all risks of delivery under bombardment. Kharkiv municipal perinatal center was damaged at the beginning of the war (Figure 1). Despite the danger, some pregnant ladies from Kharkiv took a chance and managed.

The psychological disorders during wartime could disturb female reproductive health or complicate the current of gestation. The disorders of the female reproductive system are known both in civilian and military women [20-25]. The increased level of mental disorders is involved in abnormal fetal neurodevelopment [26]. The hypothesis of fetal programming was based on the negative impact of fetal growth restriction on its health in the future lifetime [27]. The increased incidence of gestational, obstetric, and perinatal complications is typical for the female population in the area of armed conflict and for refugees [28-35]. But the variables of obstetric care during peace and wartime were almost similar in this study. The level of anemia, uterine contractile activity in labor abnormalities, and episiotomies were surprisingly lower during wartime in March.

The level of partnership in labor was lower for the first half a year. The husbands left their families to defend Ukraine from the enemy. The stressed environment was a possible reason for the elevation of cardiovascular disease and endocrine disorders amongst pregnant ladies near front line [12].

Following the evidence-based clinical protocols was the main rule that contributed to beneficial maternal and perinatal outcomes. The use of modern perinatal technologies captured the significant professional level of the staff. The absence of obstetric aggression and the stable level of normal delivery were based on the current approaches to labor induction [36, 37]. Active management was a measure for the prevention of prolonged stay in the clinic. It was not possible to collect patients in the department of feto-maternal medicine. The delayed stay in the clinic was associated with an increased risk of damage. Thus, early discharge was a measure for the reduction of traumatic injuries.

**Figure 2.**
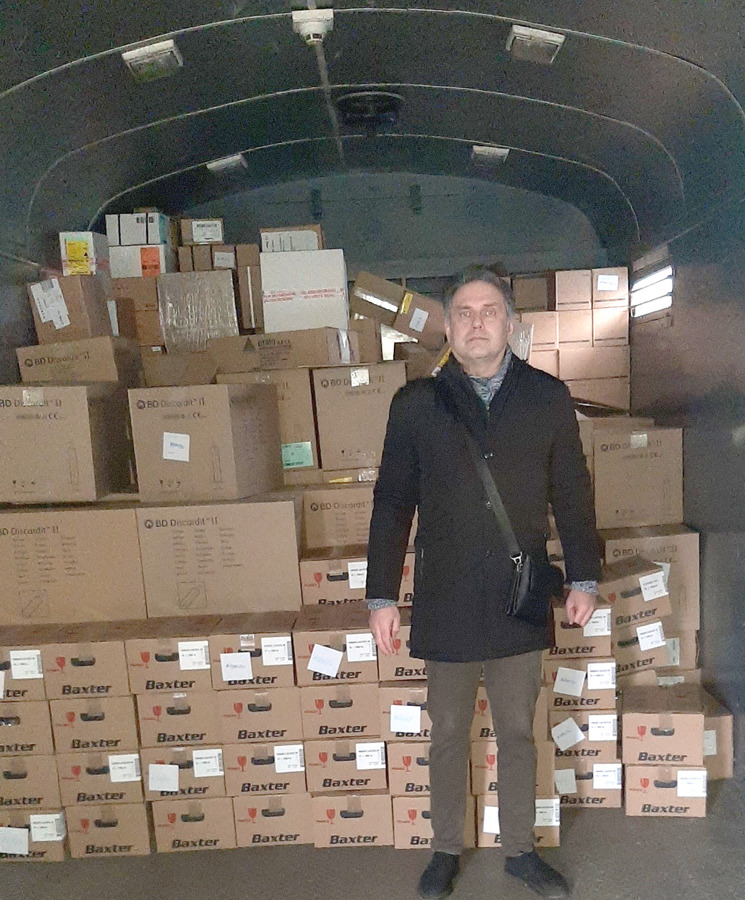
A humanitarian aid from “Doctors without borders”.

Undoubtedly, modern obstetric care requires substantial support in medicines and medical devices. Kharkiv municipal perinatal center received humanitarian aid from “Doctors without borders”. This noble action contributed enormously to the support of safe birth in Saltivka near the frontline. Our clinic was provided with all necessary medications for urgent care in a disaster setting [12].

The findings of the study could be the repercussions of the strong traditions and reserves of the obstetric community in Kharkiv. Possibly, the changes in the humanitarian situation will contribute to the shift in maternal pathologies and obstetric complications (Figure 2). Thus, further investigations are to be continued.

The paper highlighted the portrait of maternal diseases and obstetric pathology in the area of armed conflict.

The limitations of this study are a restricted period of observation and the different sizes of the study groups.

## Conclusion

The study did not reveal any significant changes in the structure of maternal pathologies and obstetric complications during the first months of wartime near the front line. But later the levels of cardiovascular disease and endocrine disorders were found to be elevated.

## References

[1] Middleton P, Shepherd E, Morris J, Crowther CA, Gomersall JC. Induction of labour at or beyond 37 weeks’ gestation. Cochrane Database Syst Rev. 2020 Jul 15;7(7):CD004945. doi:10.1002/14651858.CD004945.pub5 32666584 PMC7389871

[2] Mugo NS, Dibley MJ, Damundu EY, Alam A. “The system here isn’t on patients’ side”- perspectives of women and men on the barriers to accessing and utilizing maternal healthcare services in South Sudan. BMC Health Serv Res. 2018 Jan 9;18(1):10. doi:10.1186/s12913-017-2788-9 29316933 PMC5759875

[3] Hassan SJ, Wick L, DeJong J. A glance into the hidden burden of maternal morbidity and patterns of management in a Palestinian governmental referral hospital. Women Birth. 2015 Dec;28(4):e148-e156. doi:10.1016/j.wombi.2015.08.008 26340885

[4] Linton A, Peterson MR. Effect of managed care enrollment on primary and repeat cesarean rates among U.S. Department of Defense health care beneficiaries in military and civilian hospitals worldwide, 1999-2002. Birth. 2004 Dec;31(4):254-264. doi:10.1111/j.0730-7659.2004.00317.x 15566337

[5] Kanmaz AG, İnan AH, Beyan E, Özgür S, Budak A. Obstetric Outcomes of Syrian Refugees and Turkish Citizens. Arch Iran Med. 2019 Sep 1;22(9):482-488. 31679368

[6] Bakken KS, Skjeldal OH, Stray-Pedersen B. Immigrants from conflict-zone countries: an observational comparison study of obstetric outcomes in a low-risk maternity ward in Norway. BMC Pregnancy and Childbirth. 2015;15:163-175. doi:10.1186/s12884-015-0603-3 26243275 PMC4523905

[7] Knudson MM. Military-Civilian Partnerships to Expand Emergency Obstetric Care for Both Civilian and Military Mothers-to-Be. JAMA Netw Open. 2022;5(1):e2142843. doi:10.1001/jamanetworkopen.2021.42843 35006252

[8] Keren M, Keren N, Eden A, et al. The complex impact of five years of stress related to life-threatening events on pregnancy outcomes: a preliminary retrospective study. Eur Psychiatry. 2015;30:317-321. doi:10.1016/j.eurpsy.2014.10.004 25498241

[9] Ameh CA, Bishop S, Kongnyuy E, et al. Challenges to the provision of emergency obstetric care in Iraq. Matern Child Health J. 2011;15:4-11. doi:10.1007/s10995-009-0545-3 19946792

[10] Skokić F, Muratović S, Radoja G. Perinatal and maternal outcomes in Tuzla Canton during 1992-1995 war in Bosnia and Herzegovina. Croat Med J. 2006;47:714-721. 17042063 PMC2080464

[11] Skokić F, Bačaj D, Selimović A, et al. Association of Low Birth Weight Infants and Maternal Sociodemographic Status in Tuzla Canton during 1992-1995 War Period in Bosnia and Herzegovina. Int J Pediatr. 2010;2010:1-7. doi:10.1155/2010/789183 PMC3068307 21490700

[12] Joseph NT, Curtis BH, Goodman A. Disaster settings: Care of pregnant patients. UpToDate. 2022. https://www.uptodate.com/contents/disaster-settings-care-of-pregnant-patients

[13] Gestational Hypertension and Preeclampsia: ACOG Practice Bulletin, Number 222. Obstet Gynecol. 2020;135(6):e237-e260. doi:10.1097/AOG.0000000000003891 32443079

[14] American College of Obstetricians and Gynecologists‘ Committee on Practice Bulletins—Obstetrics. Anemia in Pregnancy: ACOG Practice Bulletin, Number 233. Obstet Gynecol. 2021 Aug 1;138(2):e55-e64. doi:10.1097/AOG.0000000000004477 34293770

[15] Chronic hypertension in pregnancy. ACOG Practice Bulletin No. 203. American College of Obstetricians and Gynecologists. Obstet Gynecol. 2019;133:e26-e50. doi:10.1097/AOG.0000000000003020 30575676

[16] ACOG Practice Bulletin No. 190: Gestational Diabetes Mellitus. Obstet Gynecol. 2018;131(2):e49-e64. doi:10.1097/AOG.0000000000002501 29370047

[17] O‘Riordan N, Robson M, McAuliffe FM. Management of poor progress in labour. Obstetrics, Gynaecology & Reproductive Medicine. 2021;31(12):342-350. 10.1016/j.ogrm.2021.10.003

[18] Committee on Practice Bulletins-Obstetrics. Practice Bulletin No. 183: Postpartum Hemorrhage. Obstet Gynecol. 2017;130(4):e168-e186. doi:10.1097/AOG.0000000000002351 28937571

[19] Southall D. Armed conflict women and girls who are pregnant, infants and children; a neglected public health challenge. What can health professionals do? Early Hum Dev. 2011 Nov;87(11):735-742. doi:10.1016/j.earlhumdev.2011.08.020 21945358

[20] Behboudi-Gandevani S, Bidhendi-Yarandi R, Panahi MH, Mardani A, Prinds C, Vaismoradi M. Perinatal and Neonatal Outcomes in Immigrants From Conflict-Zone Countries: A Systematic Review and Meta-Analysis of Observational Studies. Front Public Health. 2022;10:766943. Published 2022 Mar 11. doi:10.3389/fpubh.2022.766943 35359776 PMC8962623

[21] Bastola K, Koponen P, Härkänen T, Luoto R, Gissler M, Kinnunen TI. Delivery and its complications among women of Somali, Kurdish, and Russian origin, and women in the general population in Finland. Birth. 2019;46(1):35-41. doi:10.1111/birt.12357 29781088

[22] Bouchghoul H, Hornez E, Duval-Arnould X, Philippe HJ, Nizard J. Humanitarian obstetric care for refugees of the Syrian war. The first 6 months of experience of Gynécologie Sans Frontières in Zaatari Refugee Camp (Jordan). Acta Obstet Gynecol Scand. 2015 Jul;94(7):755-759. doi:10.1111/aogs.12638 25817053

[23] Al-Rukeimi AA, Al-Haddad A, Ali AA, Adam I. High rate of uterine rupture in a conflict setting of Hajjah, Yemen. J Obstet Gynaecol. 2017;37(8):1106-1107. doi:10.1080/01443615.2017.1324412 28760062

[24] Kang HK, Mahan CM, Lee KY, Magee CA, Mather SH, Matanoski G. Pregnancy outcomes among U.S. women Vietnam veterans. Am J Ind Med. 2000;38(4):447-454. doi:10.1002/1097-0274(200010)38:4<447::aid-ajim11>3.0.co;2-j 10982986

[25] Doyle P, Maconochie N, Davies G, et al. Miscarriage, stillbirth and congenital malformation in the offspring of UK veterans of the first Gulf war. Int J Epidemiol. 2004;33(1):74-86. doi:10.1093/ije/dyh049 15075150

[26] Campbell RK, Curtin P, Bosquet Enlow M, Brunst KJ, Wright RO, Wright RJ. Disentangling Associations Among Maternal Lifetime and Prenatal Stress, Psychological Functioning During Pregnancy, Maternal Race/Ethnicity, and Infant Negative Affectivity at Age 6 Months: A Mixtures Approach. Health Equity. 2020;4(1):489-499. Published 2020 Nov 16. doi:10.1089/heq.2020.0032 33269333 PMC7703133

[27] Hoyer D, Żebrowski J, Cysarz D, et al. Monitoring fetal maturation-objectives, techniques and indices of autonomic function. Physiol Meas. 2017;38(5):R61-R88. doi:10.1088/1361-6579/aa5fca 28186000 PMC5628752

[28] Obel J, Martin AIC, Mullahzada AW, Kremer R, Maaløe N. Resilience to maintain quality of intrapartum care in war torn Yemen: a retrospective pre-post study evaluating effects of changing birth volumes in a congested frontline hospital. BMC Pregnancy Childbirth. 2021;21(1):36. doi:10.1186/s12884-020-03507-5 33413161 PMC7791801

[29] Keasley J, Blickwedel J, Quenby S. Adverse effects of exposure to armed conflict on pregnancy: a systematic review. BMJ Glob Health. 2017 Nov 28;2(4):e000377. doi:10.1136/bmjgh-2017-000377 PMC5706483 29333283

[30] Curchoe CL, Chang TA, Trolice MP, et al. Protecting life in a time of war. J Assist Reprod Genet. 2022;39(3):555-557. doi:10.1007/s10815-022-02463-7 35344142 PMC8958475

[31] Bhandari TR, Sarma PS, Kutty VR. Utilization of maternal health care services in post-conflict Nepal. Int J Womens Health. 2015 Aug 25;7:783-790. doi:10.2147/IJWH.S90556 26346111 PMC4554403

[32] Casey SE, Isa GP, Isumbisho Mazambi E, Giuffrida MM, Jayne Kulkarni M, Perera SM. Community perceptions of the impact of war on unintended pregnancy and induced abortion in Protection of Civilian sites in Juba, South Sudan. Glob Public Health. 2021 Jul 29;1-14. doi:10.1080/17441692.2021.1959939. Online ahead of print. 34323171

[33] Casey SE, Chynoweth S, Cornier N, Gallagher M, Wheeler E. Progress and gaps in reproductive health services in three humanitarian settings: Mixed-methods case studies. Conflict and Health. 2015;9(Suppl 1):S3:1-13. doi.org/10.1186/1752-1505-9-S1-S3 25798189 PMC4331815

[34] Ellsberg M, Ovince J, Murphy M, Blackwell A, Reddy D, et al. No safe place: Prevalence and correlates of violence against conflict-affected women and girls in South Sudan. PLoS One. 2020 Oct 12;15(10):e0237965. doi:10.1371/journal.pone.0237965 33044980 PMC7549805

[35] Steven VJ, Deitch J, Dumas EF, Gallagher MC, Nzau J, et al. “Provide care for everyone please”: engaging community leaders as sexual and reproductive health advocates in North and South Kivu, Democratic Republic of the Congo. Reprod Health. 2019 Jul 8;16(1):98. doi:10.1186/s12978-019-0764-z 31286984 PMC6615084

[36] Rydahl E, Eriksen L, Juhl M. Effects of induction of labor prior to post-term in low-risk pregnancies: a systematic review. JBI Database System Rev Implement Rep. 2019 Feb;17(2):170-208. doi:10.11124/JBISRIR-2017-003587 PMC6382053 30299344

[37] Souizi B, Mortazavi F, Haeri S, Borzoee F. Comparison of vaginal misoprostol, laminaria, and isosorbide dinitrate on cervical preparation and labor duration of term parturient: a randomized double-blind clinical trial. Electron Physician. 2018 May 5;10(5):6756-6763. doi:10.19082/675 29997758 PMC6033123

